# Visualizing the quantile survival time difference curve

**DOI:** 10.1111/jep.12948

**Published:** 2018-05-23

**Authors:** Harald Heinzl, Martina Mittlboeck

**Affiliations:** ^1^ Section for Clinical Biometrics, Center for Medical Statistics, Informatics, and Intelligent Systems Medical University of Vienna Vienna Austria

**Keywords:** bootstrap samples, confidence bands, exploratory data analysis, Kaplan‐Meier curves, SAS macro

## Abstract

The difference between the *p*th quantiles of 2 survival functions can be used to compare patients' survival between 2 therapies. Setting *p* = 0.5 yields the median survival time difference. Varying *p* between 0 and 1 defines the quantile survival time difference curve which can be straightforwardly estimated by the horizontal differences between 2 Kaplan‐Meier curves. The estimate's variability can be visualized by adding either a bundle of resampled bootstrap step functions or, alternatively, approximate bootstrap confidence bands. The user‐friendly SAS software macro %kmdiff enables the straightforward application of this exploratory graphical approach. The macro is described, and its application is exemplified with breast cancer data. The advantages and limitations of the approach are discussed.

## 1 INTRODUCTION

As consistent estimator of the survival function, the Kaplan‐Meier curve^1^ is the most commonly used graphical tool in survival analysis. It is extensively used to visually compare censored survival time curves between groups of patients distinguished by different therapies, biomarker categories, or demographic features. Estimates for survival probabilities at specific times (eg, 3‐year survival probability) and specific quantiles of the survival time distribution (eg, median survival time) can be easily obtained.

The diagonal upper‐left‐to‐lower‐right nature of the plot, however, hampers the visual assessment of the difference between the *p*th quantiles of 2 Kaplan‐Meier curves. To overcome this problem, the quantile survival time difference curve is defined and straightforwardly estimated by calculating the horizontal differences between the corresponding Kaplan‐Meier curves. Two bootstrap‐based approaches are suggested to illustrate the estimate's variability; alternatively, the traditional normal approximation method or a smoothed empirical likelihood approach could have been considered as well.^2^

The quantile survival time difference curve is not to be confused with the survival probability difference curve^3^, ^4^, ^5^, ^6^ and approaches related to it.^7^, ^8^, ^9^, ^10^ Besides that, the term “quantile difference” itself is not unambiguously defined. It could also refer to the difference between the *p*th and *q*th quantiles of a single survival curve^11^, ^12^, eg, the interquartile range for *p* = 0.75 and *q* = 0.25.

In Section 2, the technical details of the approach are presented. The SAS macro %kmdiff is described and illustrated in Section 3. A brief discussion is given in Section 4. The SAS macro code was generated using Version 9.4 (for Windows) of the SAS software (copyright © 2002‐2012 SAS Institute Inc.). SAS and all other SAS Institute Inc. product or service names are registered trademarks or trademarks of SAS Institute Inc., Cary, NC, USA.

## 2 METHODS

Assume that from a large patient population with survival function *S*(*t*), right‐censored survival data have been observed for a sample of *n* patients, where *x*_1_…*x*_*n*_ denote observed survival times and *a*_1_…*a*_*n*_ denote their corresponding censoring indicators, respectively. The Kaplan‐Meier estimator of the survival function is defined as

(1) $$\hat{S}\left(t\right)=\prod_{t_{i}\leq t}\left(1-\frac{d_{i}}{n_{i}}\right),\;t_{1}\leq t\leq t_{K},$$

where 0 ≤ *t*_1_ < *t*_2_ < … < *t*_*K*_ are *K* > 0 different observed failure times, *d*_*i*_ is the number of failures, and *n*_*i*_ is the size of the risk set at *t*_*i*_, *i* = 1, …, *K*. If no censored values have been observed before *t*_1_, then *n*_1_ = *n*. $\hat{S}\left(t\right)$ is right continuous, $\hat{S}\left(t\right)=1$ for 0 ≤ *t* < *t*_1_ and $0\leq \hat{S}\left(t_{K}\right)\leq \hat{S}\left(t\right)\leq 1$ for 0 ≤ *t* ≤ *t*_*K*_. Note that $\hat{S}\left(t_{K}\right)$ equals 0 only if all censored observations occur before *t*_*K*_. If patients are still alive after *t*_*K*_, then either $\hat{S}\left(t\right)$ can be set equal to $\hat{S}\left(t_{K}\right)$ from *t*_*K*_ to the largest censoring time or $\hat{S}\left(t\right)$ can be considered not defined for *t* > *t*_*K*_. In the current manuscript, the latter definition is applied. Consequently, in the case of *K* = 0, that is no observed failure times, $\hat{S}\left(t\right)$ equals 1 for *t* = 0 and is not defined for *t* > 0.

The *p*th quantile survival time *Q*(*p*) corresponds to the labelling of the survival function axis of the Kaplan‐Meier plot and is equivalent to the common (1 − *p*)th quantile of a distribution function. For $\hat{S}\left(t_{K}\right)\leq p\leq 1$ and *K* > 0, the estimator for the *p*th quantile survival time can be defined as

(2) $$\hat{Q}\left(p\right)=\min\left(\left.t_{j}\right|\hat{S}\left(t_{j}\right)\leq p\right).$$

Now assume that from a second large patient population (independent from the first one) with survival function *S*^′^(*t*), right‐censored survival data have been observed as well for a sample of *m* patients. Their observed survival times and censoring indicators are denoted by *y*_1_…*y*_*m*_ and *b*_1_…*b*_*m*_, respectively, and 0 ≤ *u*_1_ < *u*_2_ < … < *u*_*L*_ are the *L* different observed failure times. By analogy with formulae (1) and (2), a corresponding estimator $\hat{Q^{\prime }}\left(p\right)$ for the *p*th quantile survival time *Q*^′^(*p*) is obtained, ${\hat{S}}^{\prime }\left(u_{L}\right)\leq p\leq 1$. Note that the prime in the notations refers to the second population and its corresponding sample.

Let

(3) $$p^{0}=\max\left(\hat{S}\left(t_{K}\right),{\hat{S}}^{\prime }\left(u_{L}\right)\right).$$

Now, an estimator for the quantile survival time difference curve *D*(*p*) = *Q*(*p*) − *Q*^′^(*p*) can be defined as

(4) $$\hat{D}\left(p\right)=\hat{Q}\left(p\right)-\hat{Q^{\prime }}\left(p\right)$$

for *p*^0^ ≤ *p* ≤ 1. If *p*^0^ = 0, then $\hat{D}\left(0\right)=t_{K}-u_{L}$.

It seems obvious now to plot $\hat{D}\left(p\right)$ against *p*. However, for the sake of comparability with the Kaplan Meier plot, *p* has to be plotted on the vertical axis against $\hat{D}\left(p\right)$ on the horizontal axis, respectively.

In a next step, the variability of the estimator $\hat{D}\left(p\right)$ will be visualized. For this purpose, 2 bootstrap solutions are suggested, both are based on Efron's classical bootstrap for censored data.^13^

### 2.1 Bootstrap bundle

Draw a sample of size *n* with replacement from (*x*_1_, *a*_1_), (*x*_2_, *a*_2_)…(*x*_*n*_, *a*_*n*_) and a sample of size *m* with replacement from (*y*_1_, b_1_), (*y*_2_, b_2_)…(*y*_*m*_, b_*m*_). These are the first bootstrap samples, $\left(x_{1}^{*1},a_{1}^{*1}\right),\left(x_{2}^{*1},a_{2}^{*1}\right)\ldots \left(x_{n}^{*1},a_{n}^{*1}\right)$ and $\left(y_{1}^{*1},b_{1}^{*1}\right),\left(y_{2}^{*1},b_{2}^{*1}\right)\ldots \left(y_{m}^{*1},b_{m}^{*1}\right)$ from where the bootstrapped quantile survival time difference curve can be computed from the 2 bootstrapped quantile survival time curves, $\hat{D}{*}^{1}\left(p\right)=\hat{Q}{*}^{1}\left(p\right)-\hat{Q^{\prime }}{*}^{1}\left(p\right)$, p*^1^ ≤ *p* ≤ 1. Here, p*^1^ is defined in analogy to *p*^0^ of formula (3).

Repeating the drawing of the bootstrap samples *bundle* times eventually yields *bundle* bootstrapped quantile difference curves, $\hat{D}{*}^{i}\left(p\right)$, p*^i^ ≤ *p* ≤ 1, *i* = 1…*bundle*. Depicting them together with $\hat{D}\left(p\right)$ enables a visual assessment of the variation of $\hat{D}\left(p\right)$. In doing so, a subdued colour like light grey should be used for the bootstrapped quantile difference curves, and a vibrant colour like green should be used for $\hat{D}\left(p\right)$, respectively. Setting *bundle* to a value between 40 and 200 seems reasonable when using the bootstrap bundle approach.

### 2.2 Bootstrap confidence bands

The generation of a confidence band also requires bootstrapped quantile difference curves, $\hat{D}{*}^{i}\left(p\right)$, 0 ≤ p*^i^ ≤ *p* ≤ 1, *i* = 1…*boot*. Here, the value of *boot* should depend on the chosen confidence level 100(1 − *α*)%. As rule of thumb, we recommend *boot* ≥ 100/*α*, that is *boot* ≥ 2000 for a 2‐sided 95% confidence band and *boot* ≥ 10000 for a 2‐sided 99% confidence band, respectively.

A confidence band for the quantile difference curve can be constructed from a series of pointwise confidence intervals for quantile differences. For a given survival probability *p*, the corresponding confidence interval is determined as the (*α*/2)th and the 1 − (*α*/2)th quantile of the empirical distribution of $\hat{D}{*}^{1}\left(p\right)$, $\hat{D}{*}^{2}\left(p\right)$… $\hat{D}{*}^{\text{boot}}\left(p\right)$, where p*_max_ ≤ *p* ≤ 1 and p*_max_ =  max (p*^1^, p*^2^…p*^boot^). Note that p*_max_ is the smallest probability for which the quantile difference can be computed for all bootstrap replications, and so, the confidence band is available for the interval [p*_max_, 1].

The length of this interval can become rather short as it depends on the bootstrapped Kaplan‐Meier curve (out of 2 × *boot* such curves) that drops least. By applying a conservative approach, the confidence band can now be extended for p*_1 − *α*_ ≤ *p* < p*_max_ as well, where p*_1 − *α*_ is a specifically defined (1 − *α*) quantile of p*^1^, p*^2^…p*^boot^. According to the usual definition of quantiles, the (1 − *α*) quantile is either one of the values p*^1^, p*^2^…p*^boot^ or it can be any chosen value from an interval, say [p*^j^, p*^k^]. In the former case, set $p_{1-\alpha }^{*}$ equal to that value; in the latter case, set p*_1 − *α*_ = p*^k^, respectively.

Now, for a given survival probability *p* with p*_1 − *α*_ ≤ *p* < p*_max_ and all *i* with p*^i^ ≤ *p*, $\hat{D}{*}^{i}\left(p\right)$ is defined and a weight of 1/*boot* can be assigned; furthermore, *M* is set to an arbitrary value larger than the maximum of the absolute values of these $\hat{D}{*}^{i}\left(p\right)$. For all *i* with p*^i^ > *p*, $\hat{D}{*}^{i}\left(p\right)$ is undefined and will be replaced with 2 values, −*M* and *M*, with weights 0.5/*boot*. Hence, the sum of all weights will be 1 and the confidence interval can be determined as the (*α*/2)th and the 1 − (*α*/2)th quantile of the now‐weighted empirical distribution.

## 3 RESULTS

### 3.1 SAS macro

The SAS macro %kmdiff allows the straightforward application of the method to survival data sets and is freely available at https://cemsiis.meduniwien.ac.at/en/kb/science-research/software/statistical-software.

The macro produces the standard Kaplan‐Meier plot of SAS, and plots of the quantile differences with (i) a bootstrap bundle, (ii) bootstrap confidence bands, and (iii) both a bootstrap bundle and bootstrap confidence bands. The macro parameters are described in Table 1.

**Table 1:** Parameters of the SAS macro %kmdiff

Parameter	Description
data	Input SAS data set (required); its name must not start with two underscores, and it must not contain variables whose names start with two underscores (in particular, “__g”, “__t”, and “__status”)
time	Survival time variable (required)
timeunit	Time units of the survival time variable; default is “years”
status	Survival status variable (required)
censval	Censoring status value(s) to be used in PROC LIFETEST; default is “0”; use the %str() function to specify more than 1 value
group	Covariate which distinguishes the two groups of interest (required)
gvalue1	Numerical value of first group of interest (required)
gvalue2	Numerical value of second group of interest (required)
grouplbl	Label of group variable (optional)
gvallbl1	Label of first group value (optional)
gvallbl2	Label of second group value (optional)
alpha	100‐alpha % is the pointwise two‐sided confidence level; default for alpha is “5”
boot	Number of bootstrap replications for computing confidence bands; default is “2000”, minimum is “100”
bundle	Number of bootstrap replications shown in figures; default is “200”
seedval	Bootstrap random number seed; default is “0”

### 3.2 Example

Patients with primary node positive breast cancer have been recruited by the German Breast Cancer Study Group (GBSG) from July 1984 to December 1989^14^; the GBSG data set can be either obtained from http://biostat.mc.vanderbilt.edu/wiki/Main/DataSets or from http://biom131.imbi.uni-freiburg.de/biom/Royston-Sauerbrei-book/. It will be used in the following for illustration purpose. The data set contains several clinical variables and the recurrence free survival (RFS) time of 686 female patients of whom 299 suffered an event (cancer recurrence or death).

**Figure 1:**
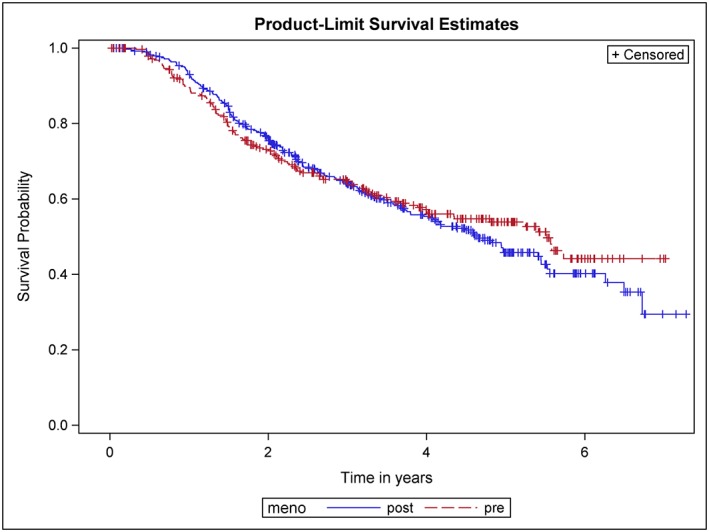
Kaplan‐Meier curves showing recurrence‐free survival (RFS) of postmenopausal and premenopausal women with primary node positive breast cancer

In Figure 1, the Kaplan‐Meier curves (product‐limit survival estimates) for 290 premenopausal and 396 postmenopausal women are shown. The curves lie close to each other and intersect right before 6 months and then again right before 3 years; albeit the first intersection is hard to see. Before the first and after the second intersection, the premenopausal women show better RFS, in the time between the postmenopausal women are better off.

**Figure 2:**
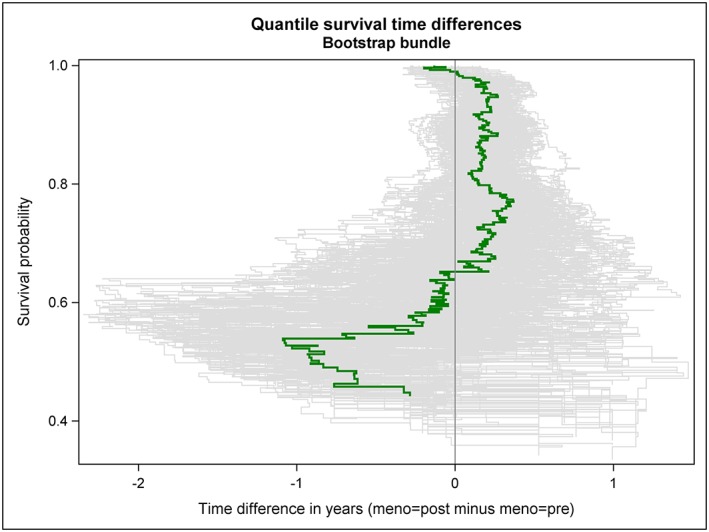
RFS probabilities are plotted against quantile RFS time differences between postmenopausal and premenopausal women with primary node positive breast cancer (green solid line). The variability of the estimated curve is illustrated by a bundle of 200 bootstrap replications (grey solid lines)

**Figure 3:**
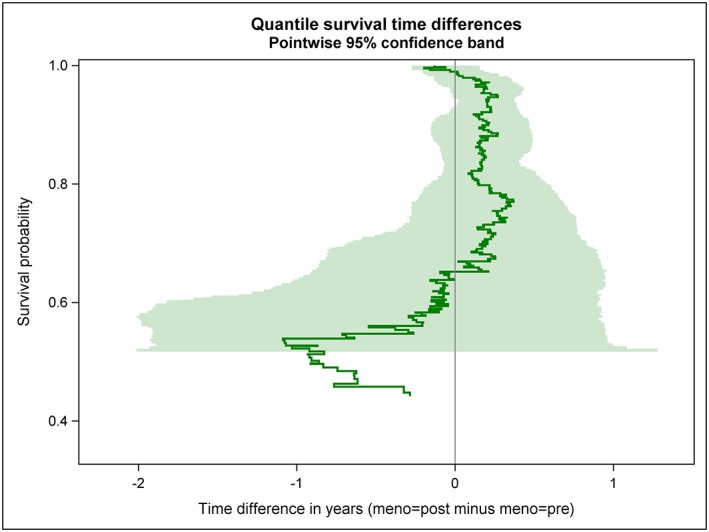
RFS probabilities are plotted against quantile RFS time differences between postmenopausal and premenopausal women with primary node positive breast cancer (green solid line). The variability of the estimated curve is illustrated by a bootstrap‐based 95% confidence band (2000 replications, green transparent area)

**Figure 4:**
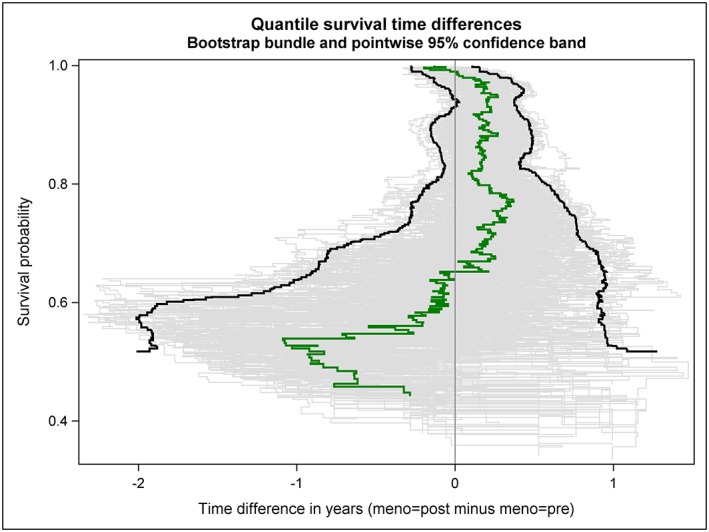
RFS probabilities are plotted against quantile RFS time differences between postmenopausal and premenopausal women with primary node positive breast cancer (green solid line). The variability of the estimated curve is illustrated by both a bundle of 200 bootstrap replications (grey solid lines) and a bootstrap‐based 95% confidence band (2000 replications, black solid lines)

The estimated quantile RFS time difference curve is shown as green solid line in Figures 2, 3, 4; thereby, 3 distinct ways to visualize its variability have been applied. A bundle of 200 bootstrap replications of the quantile RFS time difference curve are added as grey solid lines to Figure 2. A 95% confidence band is shown as green transparent area in Figure 3; it has been derived from 2000 bootstrap replications of the quantile RFS time difference curve. In Figure 4, key features of Figures 2 and 3 have been combined; however, the 95% confidence band is depicted with 2 black solid lines now. To allow independent replications of Figures 2, 3, 4, the seedval parameter (bootstrap random number seed) was set to a value larger than 0, concretely 44181.

The quantile RFS time differences curve vividly shows the extent of the horizontal gaps between the 2 Kaplan‐Meier curves. In particular, the 2 intersections of the Kaplan‐Meier curves can be easily distinguished in the quantile RFS time difference curve.

Both the bundle of bootstrap replications and the 95% confidence band reveal the increasing variability of the observed quantile difference with decreasing survival probability (ie, decreasing size of the risk set). Simple random fluctuations can provide a plausible explanation for the observed differences in quantile RFS times between premenopausal and postmenopausal women over nearly the whole observed probability range. The only exception is observed for survival probabilities around 94%.

## 4 DISCUSSION

The SAS macro %kmdiff provides an easy‐to‐use computational tool to visually assess the estimated quantile survival time difference curve and its sample variability. The curve is intended as useful complement but not as replacement of the survival probability difference curve and all the refined approaches based thereon.^3^, ^4^, ^5^, ^6^, ^7^, ^8^, ^9^, ^10^

The motivation to plot a quantile survival time difference curve may be threefold. Firstly, patients as well as health professionals may find time differences more intuitive and easier to interpret than probabilities and probability differences. Secondly, the curve shows an overall picture unlike the isolated snippet provided by the commonly reported median survival time difference. And thirdly, it can become rather difficult to assess horizontal (and also vertical) differences between 2 Kaplan‐Meier curves; the visual perception is often affected by the shortest (Euclidean) distance between the 2 curves.

The main purpose of the SAS macro %kmdiff is to support the exploration of (possibly time‐dependent) group effects in the presence of right censoring. Using the macro for confirmatory purposes would require the prespecification of statistical hypotheses and the proper adjustment for any multiple testing; it should also be taken into account that the macro produces a pointwise confidence band.

There is a potential limitation of bootstrap‐based confidence intervals for quantile differences: in particular in very small samples and risk sets, the actual coverage probability may considerably deviate from the nominal one.^15^, ^16^ Naturally, this limitation also affects the bootstrap bundle approach; that is, the graphically shown bootstrap replications might only provide a distorted picture of the variability of the estimated quantile differences in very small samples and risk sets.

Confidence intervals could also be obtained by normal approximation or a smoothed empirical likelihood method.^2^ However, the former will need moderate to large sample sizes to work properly, whereas the latter requires the selection of a kernel bandwidth by cross‐validation which is computationally burdensome.^2^ Besides that, due to the close relationship between the bootstrap and the empirical likelihood, it seems reasonable to assume that the empirical likelihood will face similar problems as the bootstrap in very small samples and risk sets.

The presented SAS macro %kmdiff only uses Base SAS and SAS/STAT procedures for statistical computations; graphical representations are based on the ODS Graphics procedure SGPLOT. Note that the user can easily modify the SGPLOT code or replace it with purpose‐built SAS code to obtain specially tailored graphical output.

In conclusion, the SAS macro %kmdiff provides a useful exploratory tool for medical researchers as it brings in an additional dimension to the assessment and communication of group differences in patient survival.
